# A Pilot Study of the Effect of a Non-Contact Boxing Exercise Intervention on Respiratory Pressure and Phonation Aerodynamics in People with Parkinson’s Disease

**DOI:** 10.3390/jcm12144806

**Published:** 2023-07-21

**Authors:** Christopher R. Watts, Zoë Thijs, Adam King, Joshua C. Carr, Ryan Porter

**Affiliations:** 1 Davies School of Communication Sciences & Disorders, Texas Christian University, Fort Worth, TX 76109, USA; 2 Department of Communication Sciences & Disorders, Molloy University, Rockville Centre, New York, NY 11570, USA; zthijs@molloy.edu; 3 Department of Kinesiology, Texas Christian University, Fort Worth, TX 76109, USA; a.king@tcu.edu (A.K.); joshua.carr@tcu.edu (J.C.C.); r.porter@tcu.edu (R.P.)

**Keywords:** dysphonia, Parkinson’s disease, respiration

## Abstract

This study investigated the effects of a non-contact boxing exercise program on maximum expiratory pressure and aerodynamic voice measurements. Methods: Eight adult males diagnosed with Parkinson’s disease participated in the study. Individuals participated in twice-weekly exercise classes lasting one hour across 12-months. Dependent variables were measured on three baseline days and then at six additional time points. A pressure meter acquired maximum expiratory pressure, and a pneumotachograph system acquired transglottal airflow and subglottal air pressure. Results: Measures of average maximum expiratory pressure significantly increased after 9- and 12- months of exercise when compared to baseline. There was an increasing trend for these measures in all participants, with a corresponding large effect size. Measures of transglottal airflow and subglottal pressure did not change over the course of 9- or 12-months, although their stability may indicate that the exercise program influenced maintenance of respiratory-phonatory coordination during voicing. Conclusions: A non-contact boxing exercise program had a significant effect on maximum expiratory pressure in people with Parkinson’s disease. The aerobic nature of the program and challenges to the respiratory muscles potentially explain the “ingredient” causing this effect. The small sample size of this pilot study necessitates future research incorporating larger and more diverse participants.

## 1. Introduction

Over 90% of all people with Parkinson’s Disease (PWPD) are affected by impairments in their vocal function (i.e., dysphonia). PD dysphonia results in changes to vocal intensity, voice quality, and communication intelligibility that negatively impact activities of daily living and quality of life. The primary intervention for PD dysphonia is intensive exercise-based voice therapy, centered on increasing vocal intensity and delivered on a short-term (e.g., four weeks) high frequency (e.g., four times per week) schedule. Impairment of respiratory physiology is also a ubiquitous finding in PWPD and can contribute to the morbidity risk associated with aspiration pneumonia in later stages of the disease [1,2]. Even in the early stages, respiratory function can show impairment when measured in the context of maximum performance tasks such as expiratory and inspiratory pressure [3,4]. Decreases in maximum inspiratory pressure (MIP) and maximum expiratory pressure (MEP) in PD are thought to be associated with underlying respiratory muscle weakness and changes to central nervous system regulation of respiratory physiology. The generation of expiratory pressures is critical in airway safety associated with deglutition (e.g., for cough reflex subsequent to laryngeal penetration with or without aspiration) and also in voice production.

Voiced sounds are created through the conversion of respiratory and aerodynamic forces into sound energy. During vocal communication, activation of the respiratory muscles represents the initiation of a coordinated process of respiratory-laryngeal-vocal tract activity leading to the generation of subglottal pressure, transglottal airflow, and phonation to produce voiced acoustic energy [5]. Subglottal pressure and transglottal airflow are negatively impacted by PD and contribute to the characteristic hypophonia exemplified by low volume and breathy voice quality [6,7]. This dysphonia can be present in mild form at disease onset, but typically transitions to greater levels of severity as the disease progresses over time and will eventually impact over 70% of all people with PD (PWPD) [8,9].

Rehabilitative interventions can be effective for treating the diminished respiratory function and dysphonia of PWPD. Strong evidence has been associated with exercise-based interventions such as LSVT LOUD and a related approach, SPEAK OUT!, both of which require multiple sets and repetitions of different voice exercises over a prescribed high-intensity schedule lasting multiple weeks [10,11,12]. Another intervention utilizing the SpeechVive prosthetic device includes a form of daily exercise consisting of oral reading for 30 consecutive minutes while the device emits noise [13]. Research has found that all three of these interventions can elicit improvement of hypophonia in PD, and both LSVT LOUD and the SpeechVive device are associated with treatment-related changes in respiratory patterns and/or the aerodynamic forces underlying phonation [13,14,15].

Despite the neurodegenerative nature of the disease, people with PD retain the ability to positively adapt to the imposed demands of exercise. Consequently, exercise may promote neuroplasticity allowing the recovery or improvement in certain motor functions [16]. Exercise-based interventions hold the potential for meaningful disease modification of PD beyond impacts on voice and speech. In animal models, acute exercise resulted in neurogenesis, increased dopamine synthesis and release, and increased dopamine in the striatum [17]. Increased corticomotor excitability, elevated levels of brain-derived neurotrophic factors, and improved striatal dopamine receptor binding potential have been reported for individuals with PD who engage in long-term exercise interventions [18]. Sustained exercise over time also appears to facilitate changes in synaptic plasticity, preservation of dopaminergic cell bodies and terminals, while also bolstering levodopa efficacy [19,20]. Collectively, the functional improvements associated with exercise suggest the presence of neuroplasticity in motor-related circuitry and the ability of the brain to learn new behaviors through modification of existing neural networks. The sum of the cellular and molecular adaptations in PWPD is ultimately expressed through adaptations in the volitional neural drive during motor activity [21]. This may explain why exercise-based voice interventions modify neuromotor control of the respiratory and laryngeal subsystems underlying sound production, and why those modifications demonstrate long-term sustainability even when the formal exercise-based intervention period ends [10].

Exercise-based voice interventions are characterized by training specificity (voice-based exercises to improve voice production) which target respiratory, phonatory, and articulatory physiology [10,22,23]. Many other exercise-based interventions for PWPD, which are not specific to voice production or respiratory support for voice, have been developed for motor rehabilitation including cycling, dance, interval training, and, recently, non-contact boxing programs [23,24,25,26]. Non-contact boxing exercise programs may be ideally suited for PWPD because they incorporate multidimensional motor challenges that target the impairments of PD, including respiratory function (i.e., sustained aerobic activity requiring exertion of the respiratory muscles), speed of movement (i.e., speed bag punching drills), balance (i.e., footwork drills), strength (i.e., resistant training incorporated into the program), executive functions (i.e., sensory awareness of body positions), and they can be adapted to the physical abilities of the individual.

Evidence has shown that voice-based exercise interventions for PWPD can have carryover or “spread” effects on swallowing function, even when swallowing is not specifically targeted [27]. However, we have limited knowledge as to whether other exercise intervention programs for PWPD, which are not specific to voice production, also demonstrate similar carryover effects on respiratory support and phonation physiology. To address this problem, the purpose of this pilot study was to investigate the effects of a non-contact boxing exercise program, called “Punching Out Parkinson’s” (PoP), on measures of maximum expiratory pressure, subglottal air pressure, and transglottal airflow in PWPD. A longitudinal case series design was employed to follow participants who were new to participating in the exercise program across nine consecutive months and then again at twelve months. Our hypotheses were that the specificity of the non-contact boxing exercise program would show direct effects on maximum respiratory pressure and also show carryover effects on measures of subglottal air pressure and transglottal airflow during voice production.

## 2. Materials and Methods

Participants: Eight men with idiopathic Parkinson’s disease (PD) served as participants for this study. All participants were diagnosed by a neurologist and were currently receiving dopamine-replacement therapy. At study onset, no participant had a neurological diagnosis other than PD, and none had participated in a non-contact boxing exercise program during the past six months. No participants were receiving speech-language therapy, all were ambulatory, and all were living at home in their communities. For inclusion, clearance by a physician to perform physical exercise was required in addition to screening with the Mini-Mental State examination (MMSE). Each participant also had to verify that they were able to attend two exercise classes per week in Fort Worth, TX. Participants were asked to schedule lab visits for assessment and measurement during times when their medication was effective (e.g., not close to the next dosage cycle).

Intervention: Participants engaged in a non-contact boxing exercise program (“Punching Out Parkinson’s”—https://punchingoutparkinsons.org/) (accessed on 15 April 2022) developed by a former world champion professional boxer and adapted to meet the needs and abilities of people with PD at different levels of physiological impairment. The methodology of this program was the same as that reported by Salvatore et al., with each exercise session organized into seven stations across 60 min, with the duration of each station approximately the same [28]. The station components included warm up and cool down, resistance training, and aerobic exercise. The specific stations were warm up including stretching, footwork, heavy bag, hand mitts (manipulated by trainer), speed bag, resistance training, and a cool-down period. Participants engaged in each session as a group, and, other than warm-up and cool-down, the order of stations was rotated between participants. Each participant completed two exercise sessions every week across 12 consecutive months for a total of 120 exercise minutes per week, 480 min per month, and 5750 min total. The aerobic exercise component of each session has been estimated by Salvatore et al. at approximately 30 min per session, or one-half of the total exercise minutes [28].

**Figure 1:**
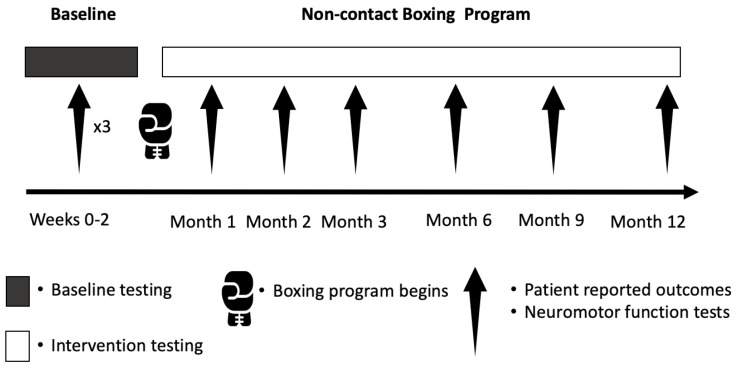
Visual form of study methodology and stages.

Measurement Schedule: The study methodology was organized into four different stages (Figure 1): baseline (pre-intervention), an intervention onset period (months 1–2), an intervention maintenance period (months 3–9), and a follow-up period (month 12). All dependent variables were measured on three different baseline days prior to the start of intervention. Once the intervention was initiated, each participant was measured during the intervention onset stage at the end of months 1 and 2. During the intervention maintenance stage, participants were measured at the end of months 3, 6, and 9, and then again at month 12 for the follow-up period. This resulted in a total of 9 unique measurement periods (3 at baseline, 2 at intervention onset, 3 at intervention maintenance, and 1 at follow-up).

Data Acquisition: Measurement sessions for data acquisition were completed in a research laboratory on a university campus on non-exercise days. An assessment battery was employed to acquire data across multiple domains. These included:

- Respiratory pressure: Respiratory support for voice production was assessed via measures of maximum expiratory pressure (MEP), in cmH_2_O, using the MicroRPM Pressure Meter (Micro Direct, Lewiston, ME). Participants were asked to maximally inhale to total lung capacity and then exhale hard and fast into the mouthpiece of the device while wearing a nose clip. Five consecutive trials were attempted. As this was a maximum performance task, the single maximum pressure from the five trials was recorded.
- Phonation (voicing) aerodynamics: Phonation aerodynamics were assessed via measures of transglottal airflow and subglottal pressure during speech tasks, using the Phonatory Aerodynamic System (PAS, Pentax Medical, Montvale, NJ, USA). For measures of transglottal airflow (in mL/s), voice waveforms were recorded while participants produced connected speech (the all-voiced sentence “We were away a year ago”) at a self-reported comfortable pitch and loudness. For measures of subglottal pressure (in cmH_2_O), participants repeated the syllable “pa” at a rate of approximately 1.5 syllables per second, at a self-reported comfortable pitch and loudness. Five trials of each transglottal airflow and subglottal pressure stimulus were recorded. The mean measurement for the five trials of each stimulus was calculated.

Analyses: Graphical visual inspection and effect size estimates were applied to the data sets of the three dependent variables separately (MEP, subglottal pressure, and transglottal airflow). For effect size, means and standard deviations of the 15 trials across the three baseline conditions were compared to the same measures of the trials across the intervention maintenance period (months 3, 6, and 9). For graphical analysis, each participant was treated as a single subject and their performance across the longitudinal study was graphed as a trend line representing that participant’s unique data set. Non-parametric Wilcoxon signed-rank tests were applied to the data sets, with an alpha level of 0.05 for statistical significance. For each dependent variable, an ad-hoc effect size analysis comparing mean baseline measurements to those at the follow-up period (month 12) was also conducted to determine continuous maintenance or improvement of any potential gains.

## 3. Results

Demographic information at study baseline associated with the eight male participants is reported in Table 1. Time since diagnosis ranged between 1 to 15 years with disease severity based on Hoehn and Yahr staging, ranging from stage 1 to stage 3. All participants were currently medicated with dopamine replacement, and none were currently enrolled in voice therapy nor had received voice therapy in the recent past. All participants were naive to the boxing exercise intervention program.

**Figure 2:**
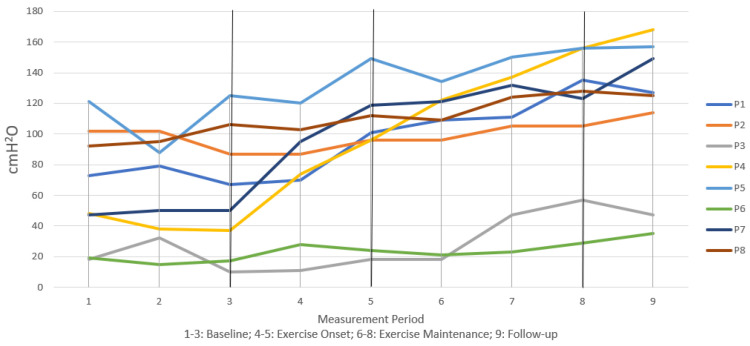
Longitudinal trends in measures of maximum expiratory pressure (MEP).

**Figure 3:**
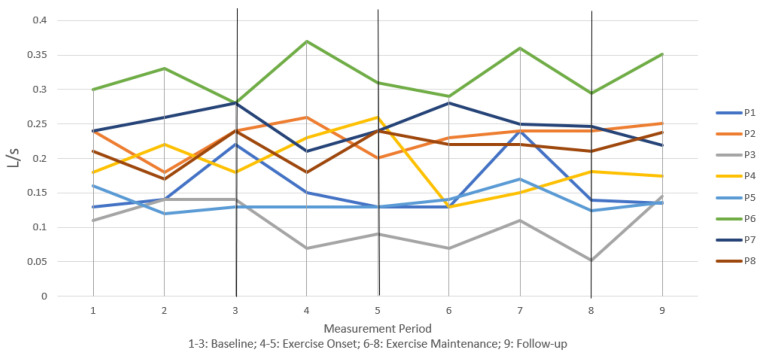
Longitudinal trends in measures of transglottal airflow.

**Figure 4:**
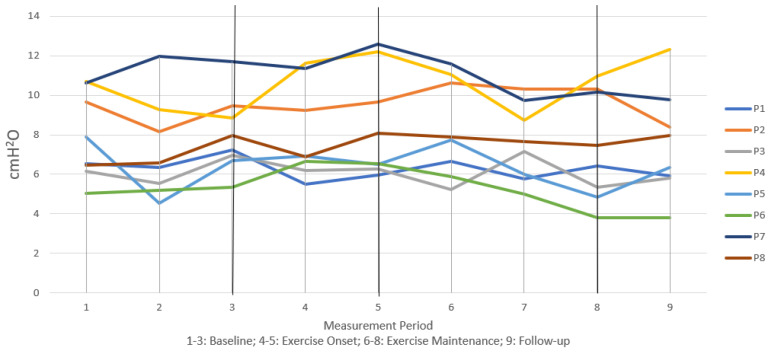
Longitudinal trends in measures of subglottal pressure.

Figure 2, Figure 3 and Figure 4 illustrate individual participant data graphed together across all measurement intervals for measures of MEP, transglottal airflow, and subglottal pressure, respectively. At the end of 9 months of regular non-contact boxing exercise, all participants demonstrated an increase in MEP and maintained those gains above baseline at follow-up (Figure 2). While baseline performance was highly variable, Figure 2 shows a clear pattern of steady increase across exercise months for most participants. Similar baseline variability was present in transglottal airflow (Figure 3) and subglottal pressure (Figure 4), without any substantial increase or decrease for individual participants. For these two variables, the graphic data suggested that baseline performance was maintained at 9 months and also at the follow-up 12-month period.

Table 2 shows effect size measurements and significance of Wilcoxon signed-rank tests. At the 9-month period there was a significant effect of exercise on MEP with a large effect size. This increase over baseline was maintained with statistical significance at the 12-month follow-up period, again with a large effect size. Effect sizes for phonation physiology measures of transglottal airflow and subglottal pressure were small and not statistically significant at either the 9-month or 12-month period. This supported the notion that phonation physiology did not change, but performance was maintained, across 12 months of exercise.

## 4. Discussion

The purpose of this pilot study was to investigate the effects of a non-contact boxing exercise program, called “Punching Out Parkinson’s” (PoP), on measures of maximum expiratory pressure and phonation physiology measures of subglottal air pressure and transglottal airflow in PWPD. At 9-months of continuous exercise at a dose of 120 min per week, we found that all participants demonstrated increases in the ability to generate MEP, and those increases were maintained at 12-months while continuing the exercise program. While there were no changes in the aerodynamics of phonation, the stability of these measures at 9-month and 12-month periods in relation to baseline abilities may be a positive finding, as phonation physiology is known to change as PD progresses over time.

The positive impact of a non-contact boxing exercise program on respiratory function may have practical significance as a non-pharmacological intervention for PD. Respiratory dysfunction in the form of inhalation and exhalation muscle weakness is a common manifestation of the disease and is strongly associated with mortality in people with PD via a connection with pneumonia [29,30]. A recent metanalysis comparing 253 PWPD to 181 controls across seven studies found significantly and substantially lower MEP in those with PD. The same metanalysis reported significantly lower measures of peak cough flow, which is associated with the ability to clear foreign material from the lower respiratory tract, in PWPD compared to controls [31]. Expected normal values of MEP in adult males is at or above 80 cmH_2_O [31,32]. Across the eight participants in the present study, average MEP at baseline was at 63 cmH_2_O and increased to over 100 cmH_2_O after 9 months of exercise, which was maintained at the 12-month period. This finding suggests that the non-contact boxing exercise program may directly address the underlying respiratory muscle weakness that is a substantial health risk factor in people with PD.

The “ingredients” of the specific exercise program studied in this investigation provides a potential explanation for the reported positive effect on respiratory function. Non-contact boxing presents an aerobic challenge to the cardiovascular system [28]. This challenge requires engagement of respiratory muscles to alter breathing cycles via faster and deeper breaths. The resistance training element specific to the PoP exercise program may have also facilitated adaptation in the respiratory muscles through increased activation of inspiratory and expiratory muscles during pushing/pulling movements. The frequency (two times per week), intensity (1-h sessions), and duration (9 months) of the PoP program was enough to elicit large increases in MEP through specific targeting of the muscles responsible for baseline respiratory weakness.

This study did not find changes to measures of phonation physiology. Both transglottal airflow and subglottal pressure varied little among the participants across the 12-month study period. While this suggests that the non-contact boxing exercise program did not have any carryover effects on voice production, it should also be noted that the PoP program was not specific to vocal function. That is, voicing and voice exercises were not a component of the exercise program, which may explain the lack of significant effect. On the other hand, it is important to note that neither measure deteriorated from baseline in any substantial way for any participant. While further research is needed to investigate this supposition, the physical challenges of the PoP exercise program may have supported maintenance of respiratory-phonatory coordination as measured with transglottal airflow and subglottal pressure. In this way, non-contact boxing may have been disease modifying for MEP (increasing motor ability) and phonation physiology (maintaining motor ability).

There are a number of limitations to this study which necessitate guarded generalizations. While the experimental design demonstrated significant increases in respiratory power associated with the exercise intervention, we did not control for other physical activities outside of the exercise program that could have also impacted respiratory function. Because exercise has been shown to consistently impact motor abilities in PWPD, activities outside of the intervention should be controlled for or considered in future studies. Each individual was also measured using the same procedures on nine different occasions, which could have facilitated learning effects and the subsequent data set. While no individuals were receiving voice therapy, we also did not control for the amount of talking/voicing that each participant engaged in during activities of daily living, and it is possible that non-voice and non-exercise activities could have influenced study results. The sample size of eight participants was very small and, although we realized strong statistical power, the sample may not be representative of the larger population of PWPD. We also only studied male participants, and whether females with PD respond in the same way to non-contact boxing exercise will need further study. In addition, we did not control for medication timing during exercise. While laboratory measurements were obtained at self-reported times of medication effectiveness, we do not know if medication timing influenced exercise activity (e.g., exertion levels) during individual sessions, and if that potential effect may have influenced results.

## 5. Conclusions

This study found that a non-contact boxing exercise program, PoP, had a significant effect on MEP in eight males with PD. While there was no effect on measures of phonation physiology, there were also no declines in those measures across the 9-month and 12-month time periods. After 9-months of exercise, the average MEP elevated from below normal thresholds to above normal thresholds, with an increasing trend of MEP was found for all eight participants. The positive results of this pilot study may inform future research seeking to investigate the effects of physical exercise on motor and non-motor abilities of people with PD.

## Figures and Tables

**Table 1:** Participant characteristics at study baseline (pre-intervention).

Participant	Current Age	Age at Diagnosis	MMSE Score	HY Stage	Dopamine Replacement	Current Speech Tx
1	66	66	25	2	Yes	No
2	64	59	30	1	Yes	No
3	82	81	28	3	Yes	No
4	74	62	27	3	Yes	No
5	64	58	28	1	Yes	No
6	63	62	30	3	Yes	No
7	66	51	30	3	Yes	No
8	62	57	25	1	Yes	No

**Table 2:** Means and standard deviations (in parentheses) for dependent variables at baseline, maintenance, and follow-up periods. Effect size data (d) are related to comparisons of baseline (mean of three baseline measurement days) to maintenance periods (mean of measurements at months 3, 6, and 9).

Dependent Variable	Baseline Days 1–3	Maintenance Months 3, 6, 9	Effect Size (d)	Significance	Follow-Up Month 12	Effect Size (d)
Respiratory Pressure (cmH_2_O)	63.25 (36.7)	101.99 (45.12)	0.94	*p* = 0.01	115.25 (49.27)	1.19
Transglottal Airflow (L/s)	0.20 (0.05)	0.19 (0.07)	0.16	*p* = 0.91	0.20 (0.07)	0.01
Subglottal Pressure (cmH_2_O)	8.03 (2.86)	7.76 (2.29)	0.10	*p* = 0.99	7.54 (2.65)	0.17

## Data Availability

The original contributions presented in the study are included in the article; further inquiries can be directed to the corresponding author.
